# Rapid Estimation of Astaxanthin and the Carotenoid-to-Chlorophyll Ratio in the Green Microalga *Chromochloris zofingiensis* Using Flow Cytometry

**DOI:** 10.3390/md15070231

**Published:** 2017-07-19

**Authors:** Junhui Chen, Dong Wei, Georg Pohnert

**Affiliations:** 1 School of Food Sciences and Engineering, South China University of Technology, Guangzhou 510641, China; jhchens@126.com; 2 Institute for Inorganic and Analytical Chemistry, Bioorganic Analytics, Friedrich Schiller University Jena, Lessingstr. 8, D-07743 Jena, Germany; Georg.pohnert@uni-jena.de

**Keywords:** astaxanthin, *Chromochloris zofingiensis*, rapid estimation, fluorescence, flow cytometry, HPLC

## Abstract

The green microalga *Chromochloris zofingiensis* can accumulate significant amounts of valuable carotenoids, mainly natural astaxanthin, a product with applications in functional food, cosmetics, nutraceuticals, and with potential therapeutic value in cardiovascular and neurological diseases. To optimize the production of astaxanthin, it is essential to monitor the content of astaxanthin in algal cells during cultivation. The widely used HPLC (high-performance liquid chromatography) method for quantitative astaxanthin determination is time-consuming and laborious. In the present work, we present a method using flow cytometry (FCM) for in vivo determination of the astaxanthin content and the carotenoid-to-chlorophyll ratio (Car/Chl) in mixotrophic *C. zofingiensis*. The method is based on the assessment of fluorescent characteristics of cellular pigments. The mean fluorescence intensity (MFI) of living cells was determined by FCM to monitor pigment formation based on the correlation between MFI detected in particular channels (FL1: 533 ± 15 nm; FL2: 585 ± 20 nm; FL3: >670 nm) and pigment content in algal cells. Through correlation and regression analysis, a linear relationship was observed between MFI in FL2 (band-pass filter, emission at 585 nm in FCM) and astaxanthin content (in HPLC) and applied for predicting astaxanthin content. With similar procedures, the relationships between MFI in different channels and Car/Chl ratio in mixotrophic *C. zofingiensis* were also determined. Car/Chl ratios could be estimated by the ratios of MFI (FL1/FL3, FL2/FL3). FCM is thus a highly efficient and feasible method for rapid estimation of astaxanthin content in the green microalga *C. zofingiensis*. The rapid FCM method is complementary to the current HPLC method, especially for rapid evaluation and prediction of astaxanthin formation as it is required during the high-throughput culture in the laboratory and mass cultivation in industry.

## 1. Introduction

Astaxanthin (3,3′-dihydroxy-β,β-carotene-4,4′-dione) is a well-known red keto-carotenoid with high commercial value in functional food, cosmetics and nutraceuticals industry because of its high anti-oxidative activities [1]. Astaxanthin is a potential therapeutic agent for cardiovascular and neurological diseases owing to its anti-oxidative, anti-inflammatory and anti-apoptotic effects [2,3]. Natural astaxanthin prevalently has (3*S*,3’*S*) stereochemistry, while synthetic astaxanthin is composed of (3*S*,3’*S*), (3*RS*,3’*RS*) and (3*R*,3’*R*) stereoisomers. The natural product is thus a superior source for highly defined additives to food and pharmaceutics [4,5]. Both the free astaxanthin and its esters from *Haematococcus pluvialis* exhibit multiple bioactivities including anti-cancer, anti-oxidation, anti-inflammation and anti-diabetes activities and cardiovascular prevention [1]. Also, the microalgal powder of *H. pluvialis* has been associated with anti-aging potential [6]. The green microalga *H. pluvialis* is the best source for the commercial production of astaxanthin followed by the red yeast *Phaffia rhodozyma* [7].

Recently, the unicellular green microalga *Chromochloris zofingiensis*, which was renamed from *Chlorella zofingiensis* [8], is considered an alternative promising producer of natural astaxanthin with the potential to replace *H. pluvialis*. It is superior to *H. pluvialis* since *C. zofingiensis* has a high growth rate and it is easy to achieve high cell density in the presence of organic substrates [9]. It accumulates astaxanthin under stress conditions [10]. Other secondary carotenoids such as keto-lutein, adonixanthin, and canthaxanthin generally formed from primary carotenoids, also remarkably accumulate in algal cells as a strategy to reduce photo-damage and to adapt to changing light conditions along with the degradation of chlorophylls and primary carotenoids [11]. Accordingly, the carotenoid-to-chlorophyll ratio (Car/Chl), as the preferable indicator of carotenogenesis in microalgae [12], increases markedly under the combined stress of high irradiance and nitrogen deprivation due to the accumulation of secondary carotenoids. Easier cell-wall disruption and higher recovery of valuable pigments make *C. zofingiensis* more attractive for astaxanthin production than *H. pluvialis* [13]. However, the content or production of astaxanthin from *C. zofingiensis* is still much lower than that of *H. pluvialis* [10]. Strategies to enhance astaxanthin accumulation in *C. zofingiensis* are still under investigation [9,14].

A fast screening method for the formation of this high-value product is required to enable carotenoid evaluation and control the production of astaxanthin. Rapid and accurate methods for the relative and absolute astaxanthin quantification in living cells are thus crucial in the high-throughput screening of cultures. HPLC methods involving C18 or C30 columns for the separation of cellular pigments are commonly used, but these protocols are time-consuming and require extraction of the cells before measurement [15]. Some rapid methods including Raman spectroscopy, near-infrared spectroscopy (NIRS) and nuclear magnetic resonance spectroscopy (NMR) are feasible for the quantitative measurement of natural carotenoids [15,16,17,18,19]; however, these methods demand expensive and specialized equipment and often do not fulfill the requirements for high-throughput screening. The fluorescence spectroscopy-based investigation is a widely used technology that is convenient for carotenoid analysis with acceptable sensitivity and accuracy.

Flow cytometry (FCM) is a powerful technology for single cell analysis through detecting forward- and side-scattered light and fluorescence signals in living cells. To date, FCM has been widely applied in the eco-physiological studies of unicellular microalgae, especially in the analysis of cellular pigments (chlorophylls, carotenoids and phycobiliproteins) based on their auto-fluorescence in real time without losing accuracy [20]. It is speculated that the auto-fluorescence of astaxanthin has the potential use to measure the content using FCM without applying additional fluorescent dyes. FCM-based methods for rapid assay and screening have been reported recently to replace the conventional methods for carotenoids and astaxanthin quantification in yeasts and the green microalga *H. pluvialis* [21,22,23]. The interferences of auto-fluorescence between carotenoids and chlorophylls are highlighted as major reasons for the limited application of FCM in pigment quantification [22].

In the present work, we present a simple FCM method to estimate the astaxanthin content in living cells of *C. zofingiensis* as a preferably complementary method to HPLC, especially for rapid evaluation and prediction of astaxanthin formation in living algal cells during high-throughput cultivation. The method allows for the relative quantification of the astaxanthin content and the Car/Chl ratio. Based on the correlation of the fluorescence intensity of algal cells and cellular pigment content measured by HPLC, the FCM method can potentially, after calibration, be used for the absolute quantification as well. The cells were first cultivated mixotrophically under the stress condition of a high C/N ratio to induce astaxanthin accumulation. Additionally, the fluorescence (FL1-FL3) of the stressed cells was monitored by FCM, followed by the measurement of astaxanthin content in frozen dried biomass using an established HPLC method. Finally, the FCM method, based on the close correlation of fluorescence intensities with carotenoid quantities determined by HPLC, was established to rapidly evaluate the astaxanthin content and Car/Chl ratio in living cells. The interferences of other carotenoids to astaxanthin quantification, recovery and repeatability of the FCM method are discussed in detail.

## 2. Results and Discussion

### 2.1. Cell Growth and Pigment Accumulation

**Figure 1:**
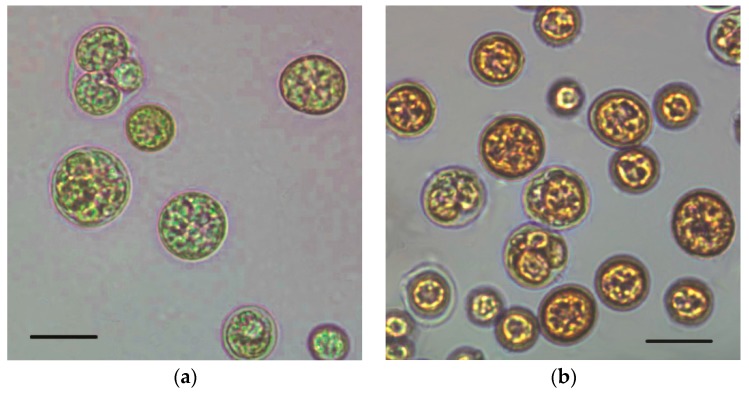
Microscopic images of *C. zofingiensis* cells during mixotrophic growth. (**a**) Initial stage at 0 h; (**b**) Final stage at 288 h; Bars: 10 μm. The mixotrophic growth experiment was performed in modified basal medium with 30 g/L of glucose and 0.6 g/L of NaNO_3_ with a high C/N ratio of 200. A light intensity at 80 μmol photon m^−2^ s^−1^ was employed for astaxanthin accumulation after inoculation of seed culture grown mixotrophically under 10 μmol photon m^−2^ s^−1^.

**Figure 2:**
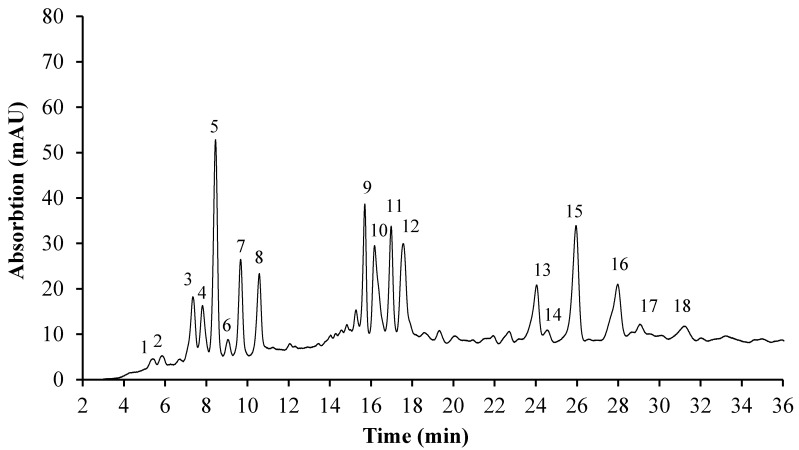
Pigment composition of the mixotrophic *C. zofingiensis* cells at 288 h determined by HPLC at 480 nm. Tentatively identified peaks: 1. Violaxanthin; 2. Neoxanthin; 3. Chlorophyll *b*; 4. Keto-lutein; 5. Lutein; 6. Astaxanthin; 7. Chlorophyll *a*; 8. Canthaxanthin; 9, 11. Keto-lutein mono-esters; 10. Astaxanthin mono-ester; 12. Carotene-like carotenoid; 13–18. Astaxanthin di-esters.

**Figure 3:**
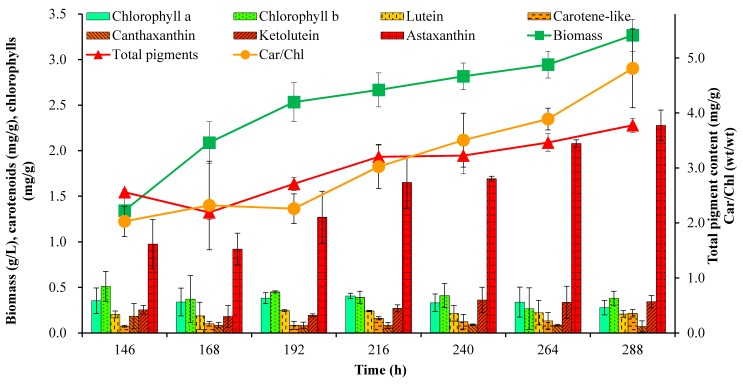
Algal biomass, pigment cell content and Car/Chl variation determined by HPLC in the culture of *C. zofingiensis*.

The green microalga *C. zofingiensis* has a high growth rate and shows substantial astaxanthin accumulation during unfavorable growth conditions such as oxidative stress, high light and nutrient starvation [10,24]. In the present work, the mixotrophic cells were non-motile and spherical with different sizes (Figure 1). Small autospores were asexually produced from large parental cells during the culture. Cells started to accumulate carotenoids, mainly astaxanthin, as the response to unfavorable conditions of high irradiation and nitrogen starvation for survival. Therefore, the cell color started to become orange or red gradually from green during the surveyed 288 h of culturing. The larger cells were obviously redder than others at the end of the cultivation (288 h), where the highest cellular astaxanthin content was observed (Figure 2 and Figure 3).

Biomass concentration and pigment composition were analyzed daily from the sixth day onwards. The pigments were analyzed by HPLC and identified by comparing the retention times and the UV-vis absorption and spectral fine structures with the standards and information from literature. As shown in Figure 2, astaxanthin and its derivatives and precursors, keto-lutein, lutein, canthaxanthin and chlorophylls were the main pigments in the stressed cells at the end of the cultivation (288 h), with similar chromatographic profiles as reported previously [10,11]. Three forms of astaxanthin (free, mono-ester and di-esters) were detected in the HPLC chromatogram (Figure 2), indicating that astaxanthin primarily existed as di-esters, while keto-lutein was mainly present in the form of the mono-ester. The other carotenoids existed predominantly in the free form.

**Figure 4:**
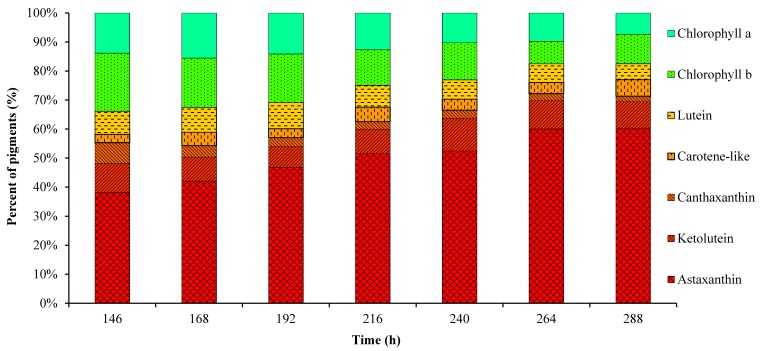
Percentage (%, total pigments) of pigments determined by HPLC in the mixotrophic cells of *C. zofingiensis*.

Figure 3 shows the variation of biomass, pigment content and Car/Chl ratio in mixotrophic cultures. Algal cells grew rapidly during the first 192 h; then, the specific growth rate decreased gradually due to limited nitrogen supply, and the final accumulated biomass was only 3.27 g/L. The astaxanthin content began to increase remarkably when the cells entered the stationary growth phase after 192 h of the culture in response to low nitrogen and high irradiation stress. In general, chlorophylls and primary carotenoids such as neoxanthin, violaxanthin and lutein, are mainly located in chloroplasts of green algae. Primary carotenoids are associated with chlorophylls in the collection of light energy and transfer the energy to chlorophylls for photosynthesis [25]. Nitrogen deficiency and high light intensity as the stress conditions led to the accumulation of secondary carotenoids such as astaxanthin and keto-lutein to resist the excess light damage along with the active degradation of chlorophylls and primary carotenoids [11]. Astaxanthin and its esters were reported as the major specific carotenoids existing in *C. zofingiensis* cells [10]. In this work, under the stress conditions applied, the astaxanthin content increased in a more pronounced manner than the other pigments, and reached the maximum of 2.3 mg/g at 288 h (Figure 3), which was consistent with a previous study [11]. Keto-lutein increased from 0.25 to 0.34 mg/g in *C. zofingiensis* cells, while the other pigments such as lutein, chlorophyll *a* and *b* changed slightly during the culture (Figure 3). As a result of the increase in the cellular astaxanthin content, the Car/Chl ratio and total pigment content increased over time, and finally reached values as high as 4.81 and 3.78 mg/g, respectively. The percentage of astaxanthin relative to all pigments increased from 38.1% at 146 h to the maximum of 60.3% at 288 h (Figure 4), which was slightly higher than the value of 50% previously reported for *C. zofingiensis* growing under unfavorable conditions [10]. Keto-lutein at 9.1% of total pigments was found as the second most abundant carotenoid after astaxanthin. The percentage of chlorophylls (*a* + *b*) relative to all pigments decreased from 31.9% at 146 h to 17.5% at 288 h (Figure 4). Overall, the changes in pigment composition of *C. zofingiensis* led to the shift in color from green to red, indicating that the stress of nitrogen-depletion and exposure to high irradiation resulted in a decrease in chlorophylls and primary carotenoids and the accumulation of secondary carotenoids, especially astaxanthin and keto-lutein.

### 2.2. Fluorescence Images and Flow Cytometric Analysis

**Figure 5:**
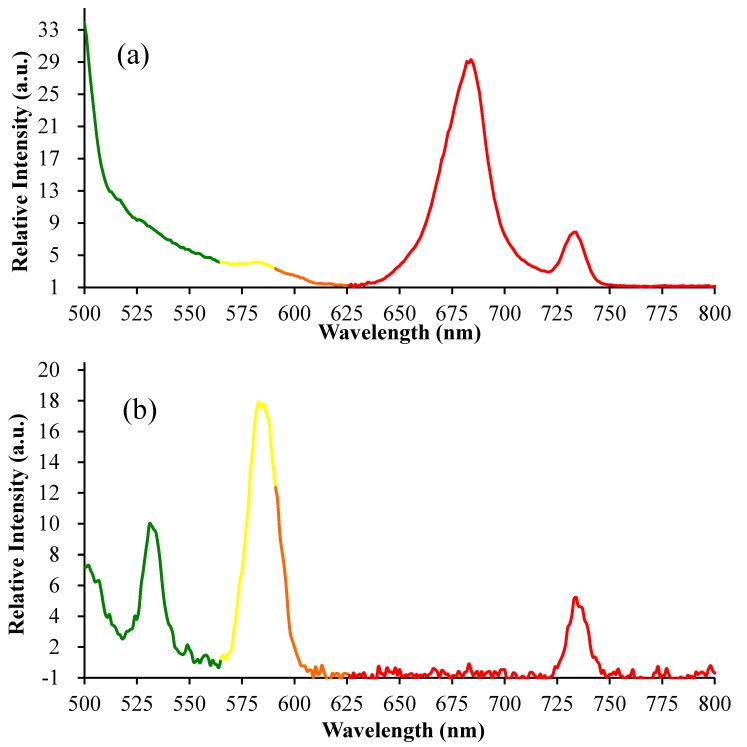
The fluorescence emission spectra of chlorophyll-rich green cells and astaxanthin-rich red cells of mixotrophic *Chromochloris zofingiensis* using a spectrofluorometer (488 nm excitation). (**a**) Chlorophyll-rich cells at 0 h; (**b**) Astaxanthin-rich cells at 288 h.

The fluorescence emission spectra of the suspension of chlorophyll-rich green cells and astaxanthin-rich red cells in pure water were measured using a spectrofluorometer (488 nm excitation) as shown in Figure 5. The mixotrophic cells emitted green, yellow, and red fluorescence with different intensities, and the fluorescence intensities changed during the stress-induced carotenogenesis. Considering that photosynthetic pigments such as chlorophylls and carotenoids are the important fluorescent compounds in biological systems [26], the changes in auto-fluorescence of algal cells might be correlated with cellular pigment composition. In green algal cells, chlorophyll *a*, chlorophyll *b* and lutein are the main pigments, while small amounts of other carotenoids, such as neoxanthin, violaxanthin, astaxanthin, ketolutein and canthaxanthin, also exist in green algal cells [11]. According to previous studies [26,27,28], chlorophyll *a* emits maximum red fluorescence at about 680 nm and low far-red fluorescence at 735 nm, and these fluorescence intensities correlated with the content of chlorophyll *a*, which was in accordance with our study as shown in Figure 5a. The red fluorescence at 680 nm could be reabsorbed by in vivo chlorophyll *a* of cells; therefore, the weak red fluorescence might be undetected, which could explain the phenomenon that we only detected the weak far-red fluorescence of algal cells in Figure 5b.

**Figure 6:**
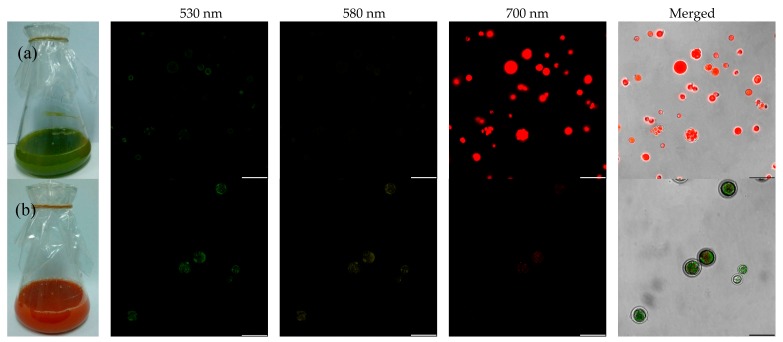
Fluorescence images of mixotrophic *C. zofingiensis* based on confocal laser scanning microscopy (CLSM). (**a**) Chlorophyll-rich cells at 0 h; (**b**) Astaxanthin-rich cells at 288 h (excitation at 488 nm, emission at 530 nm ± 10 nm, 580 nm ± 10 nm, 700 nm ± 30 nm). Bars: 25 μm.

**Figure 7:**
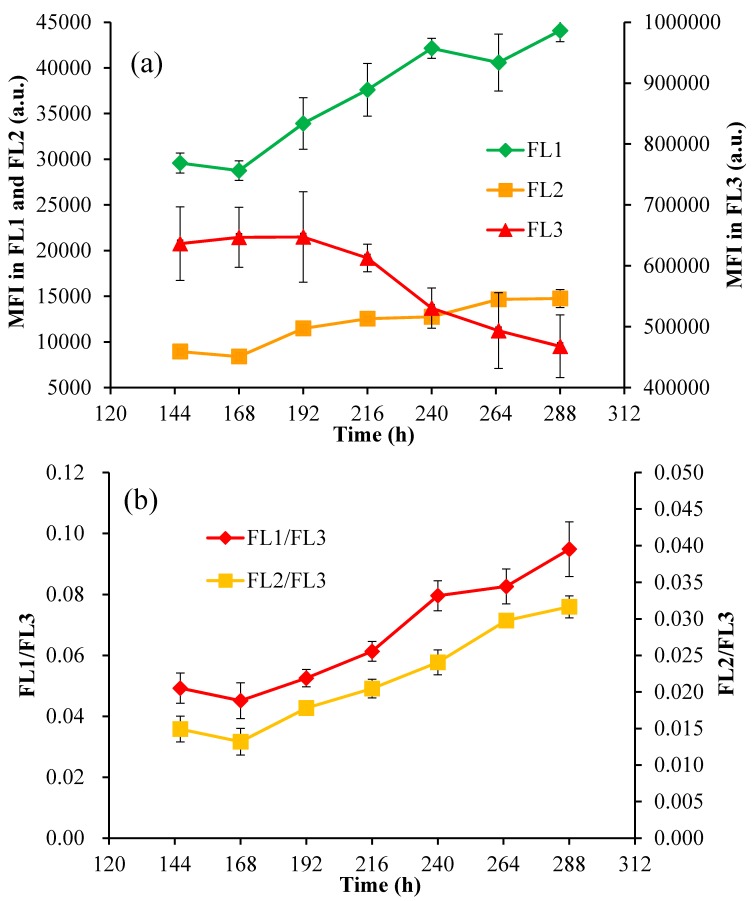
(**a**) The mean fluorescence intensities (MFI) in the FL1, FL2, and FL3 channels; (**b**) The MFI ratios of FL1/FL3 and FL2/FL3 in mixotrophic cells of *C. zofingiensis*. FL1: 533 nm ± 15 nm; FL2: 585 nm ± 20 nm; FL3: >670 nm; a.u.: arbitrary units.

As for the astaxanthin-rich red cells, their pigment composition, as shown in Figure 3 and Figure 4, was significantly different from that of green algal cells. Considering that astaxanthin, accounting for 60.3% of total pigments, was the main fluorescent compound in algal cells, the changes in auto-fluorescence of algal cells and their intensities at specific wavelengths (530 nm & 580 nm), as shown in Figure 5, Figure 6 and Figure 7, might have potential relationships with these pigments, especially astaxanthin. According to previous studies [26,27,28], the emission wavelength of 530 nm was probably caused by carotenoids, while the origin of the yellow fluorescence at 570 nm was not certain. In a previous study, the maximum excitation and emission wavelengths of astaxanthin in acetone by fluorescence analysis were 350 and 570 nm, respectively [22]. To our knowledge, the fluorescence emission spectrum of in vivo astaxanthin in algal cells has not been reported. In the microalga *H. pluvialis*, yellow fluorescence at 575 ± 15 nm (488 nm excitation) [29] and 600 ± 40 nm (546 nm excitation) [30] was used for astaxanthin analysis, although these references did not contain detailed experimental evidence of the fluorescence originating from in vivo astaxanthin. Considering that algal cells can simultaneously synthesize various carotenoids and their precursors with a similar chemical structure, it is not possible to obtain the fluorescence emission spectrum of in vivo astaxanthin due to the serious interference of auto-fluorescence between astaxanthin and other carotenoids. However, in our current work, astaxanthin, accounting for 60.3% of total pigments, was the important fluorescent compound in algal cells; thus, its fluorescence would be surely reflected in the fluorescence emission spectra of algal cells. Generally, carotenoid fluorescence is mainly attributed to the presence of the symmetrical tetraterpene skeleton formed by the conjugation of two geranylgeranyl diphosphate molecules, which is the basic structure of various carotenoids [31]. The conjugation length and chemical modification in the circuit of electrons along the conjugated bonds has been reported to affect the absorbance spectrum of carotenoids [31,32]. Thus, we assumed that the yellow fluorescence mainly originated from the series of conjugated double bonds of carotenoids and could be further affected by chemical modification of in vivo carotenoids such as esterification with different fatty acids in lipid droplets. Astaxanthin fluorescence may be also reabsorbed or disturbed by other carotenoid derivatives and precursors due to their similar structures including the symmetrical tetraterpene skeleton. Additionally, it is worth mentioning that the quantum yield of fluorescence emission depends not only on the properties of the fluorescing chromophore but also on its surroundings, which significantly influence the quenching of excitation energy [33,34]. Although there is no consensus about the origins of the green (530 nm) and yellow (570 nm) fluorescence of algal cells at present, based on our results as shown in Figure 3, Figure 4, Figure 5 and Figure 6, we propose that the changes in the fluorescence emission spectrum of algal cells in the present study were caused by the significant accumulation of secondary carotenoids, mainly astaxanthin during stress-induced carotenogenesis.

Confocal laser scanning microscopy (CLSM) was employed to visualize the cellular distribution of pigments as shown in Figure 6. Fluorescence images of *C. zofingiensis* showed that chlorophyll-rich cells emitted very strong red fluorescence, whereas astaxanthin-rich cells exhibited both green and yellow fluorescence. The changes in auto-fluorescence were closely related to the pigment composition of algal cells at different stages of the stressed culture, which could be applied to predict pigment formation. Therefore, in the following experiments, the fluorescence at approximately 530, 580, and 700 nm was selected for FCM analysis.

In vivo auto-fluorescence of the mixotrophic cells (referred to as the mean fluorescence intensities, MFI, in three channels: FL1 at 533 ± 15 nm, FL2 at 585 ± 20 nm, and FL3 > 670 nm) was recorded by FCM. MFI in FL1 and FL2 increased noticeably over the time course of the experiment, while MFI in FL3 kept on decreasing in the stressed culture (Figure 7). Accordingly, the MFI ratios of FL1 to FL3 (FL1/FL3) and FL2 to FL3 (FL2/FL3) both increased, from 0.049 to 0.095 and 0.015 to 0.032, respectively, during the stressed cultivation. Several previous studies have demonstrated that the fluorescence detected by FL3 (675 ± 20 nm) could be used to monitor the content of chlorophylls when excited at 488 nm [35], and FCM could be applied to measure the relative content of chlorophyll *a* and *b* in *Dunaliella viridis* cells by determining the MFI in FL3 and FL4 (675 ± 25 nm), respectively [36]. The yellow fluorescence at 575 ± 15 nm (488 nm excitation) [29] and 600 ± 40 nm (546 nm excitation) [30] was applied for the analysis of astaxanthin in *H. pluvialis*. Furthermore, it was reported that the chlorophyll fluorescence ratio of F684/F735 could supply useful information for the rapid estimation of cellular stress in the algal photosynthetic apparatus [37]. Thus, the MFI ratios of FL1/FL3 and FL2/FL3, which kept on increasing during carotenogenesis in the present study, may be used to investigate the cellular stress in algal cells. In *C. zofingiensis* cells, photosynthetic pigments such as chlorophylls and astaxanthin are the main fluorescent compounds. Under environmental stresses, the accumulation of secondary carotenoids was reported to co-occur with the degradation of chlorophylls under environmental stress [11], which could further affect the auto-fluorescence of algal cells. The appropriate interpretation of these auto-fluorescences and their intensities at specific wavelengths may give useful information about the presence of specific pigments and the physiological states of algal cells [26].

**Figure 8:**
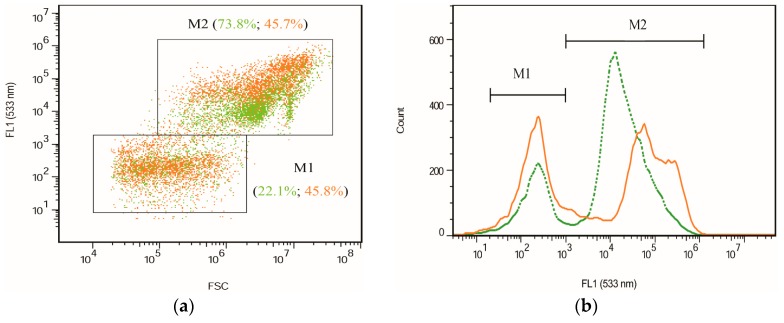

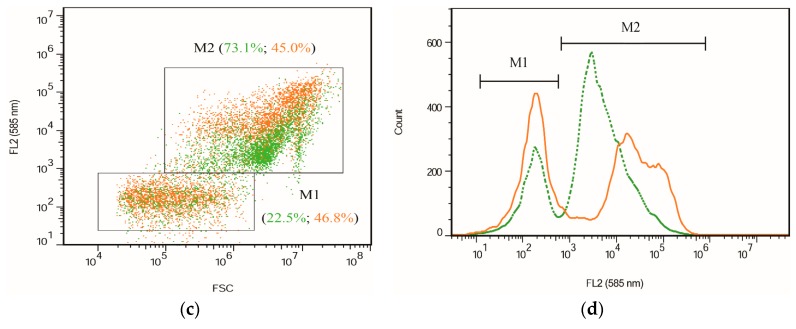
The cytograms of mixotrophic *C. zofingiensis* during cultivation based on FCM analysis. (**a**) FSC vs. FL1; (**b**) Cell count vs. FL1 (533 nm ± 15 nm); (**c**) FSC vs. FL2; and (**d**) Cell count vs. FL2 (585 nm ± 20 nm). Green dotted lines (or dots) represent the initial cells (the start of culture); orange solid lines (or dots) represent the final cells (the end of culture). M1 represents the population of small cells with low fluorescence; M2 corresponds to the population of large cells with high fluorescence.

Two different cell size populations were present in the cultures as indicated by the signals of forward scatter (FSC) (Figure 8). These two subpopulations of cells are in accordance with the microscopic observation and CLSM images. The stress of nitrogen deprivation together with high light irradiation resulted in the increase in the percentage of small cells (M1) from ca. 22% to 46% after the cultivation (Figure 8a,c), indicating that the stressed conditions resulted in the large parental cells having comparatively thin cell walls and releasing more small autospores. This was also consistent with a previous study which reported that light could promote algae proliferation and accelerate cell division in a culture under light [38]. Moreover, the cytometric results showed that small cells (M1) emitting weak fluorescence exhibited no significant difference in the fluorescence intensities in both FL1 and FL2 compared to the small cells before stressing of the culture. Conversely, the large cells (M2) emitted fluorescence strongly in both FL1 and FL2 at the end of the cultivation, although the percentage of large cells significantly decreased to approximately 45%. It is known that during the life cycle of *C. zofingiensis*, the vegetative cells contain more chlorophylls and less astaxanthin under favorable conditions, whereas under adverse growth conditions, they turn into large red cells in combination with the high accumulation of carotenoids, mainly astaxanthin, and the degradation of chlorophylls [11]. Carotenoid accumulation in large cells is consistent with the changes in fluorescence intensity based on FCM. Accordingly, the fluorescence intensities indicated by FCM could be clearly applied for the relative quantitative determination of certain cellular pigments.

### 2.3. Linear Regression of the Astaxanthin Content *versus* Fluorescence Intensities

**Figure 9:**
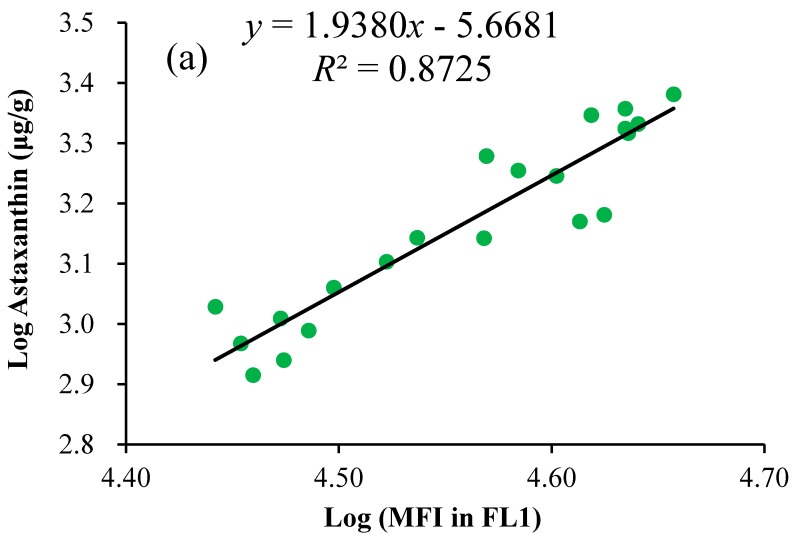

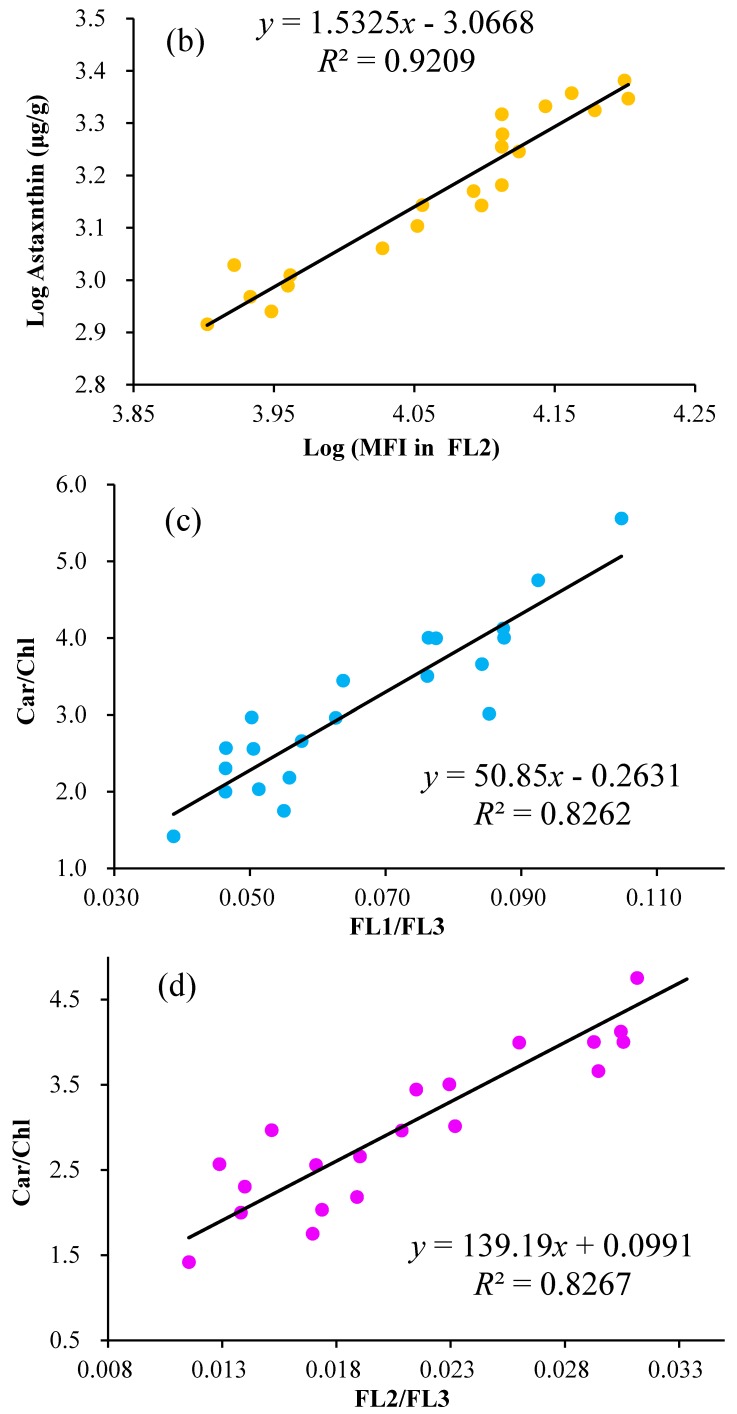
Linear relationships of astaxanthin contents versus mean fluorescence intensities (MFI) at FL1 (**a**) and FL2; (**b**), and linear relationships of Car/Chl ratios versus the ratios of FL1/FL3; (**c**) and FL2/FL3; (**d**) using FCM. Each dot represents the astaxanthin content (μg/g, dry weight) determined by HPLC and MFI by FCM from the same sample. The values of mean fluorescence intensity (a.u.) and the astaxanthin content (μg/g, dry weight) of algal cells were logarithmically transformed (log10) to improve the fit and to equalize variances.

Assuming that the auto-fluorescence of algal cells at specific wavelengths (530 nm & 580 nm) correlated positively with the cellular pigments, the relationships between fluorescence intensities in FL1 and FL2 measured by FCM and the cellular astaxanthin content measured by HPLC were explored and compared as shown in Figure 9. Considering that the values of fluorescence intensity were several orders of magnitude higher than those of the pigment content, the variables were logarithmically transformed to satisfy the homogeneity of variances assumption for the errors and linearize the fit as much as possible. Then, the astaxanthin content from HPLC measurements (μg/g) was plotted against the mean fluorescence intensity (MFI) in the channel FL1 or FL2. The correlation coefficient between the astaxanthin content and MFI in FL2 was obtained at 0.96 (*n* = 21, *p* < 0.01), which was slightly higher than the value of 0.93 (*n* = 21, *p* < 0.01) between the astaxanthin content and MFI in FL1. Furthermore, the correlation coefficients between Car/Chl ratios and the ratios of FL1/FL3 or FL2/FL3 were calculated using similar procedures, and these values both reached 0.91 (*n* = 21, *p* < 0.01). The value of the correlation coefficient higher than 0.90 indicated that strong positive correlation existed between these variables, which was consistent with our results presented above. In order to predict the astaxanthin content based on fluorescence intensity in FL1 or FL2 by FCM, regression analysis was applied to find the best fit of astaxanthin evaluation, and the linear regression was estimated through the coefficient of determination (*R*^2^). The best linear regression curve was selected for the rapid determination of astaxanthin. Furthermore, the relationship between the ratios of FL1/FL3 or FL2/FL3 and Car/Chl ratios was established by similar procedures. The FCM method with the established regression curves was then used for predicting the astaxanthin content and Car/Chl ratio of unknown samples under specific conditions through directly detecting the fluorescence intensities of unknown samples.

As shown in Figure 9a,b, the astaxanthin content was positively correlated with MFI in both FL1 and FL2. Among these, MFI in FL2 was closely correlated with the astaxanthin content (*R*^2^ = 0.92) by comparison to that of FL1 (*R*^2^ = 0.87). This was in accordance with the fluorescence emission maximum of algal cells (Figure 5). Our results indicated that MFI in FL2 was feasible to estimate the cellular astaxanthin content by the regression equation (log_10_ (astaxanthin (μg/g)) = 1.53 × log_10_ (MFI in FL2 (a.u.)) + 3.07 (*R*^2^ = 0.92)). The coefficient of determination, as high as 0.92, demonstrated that FCM correlated well with the HPLC values and could be directly applied to predict the astaxanthin content.

In a previous study, the auto-fluorescence intensity around 675 nm based on FCM showed good correlation with the actual content of astaxanthin (0.10–0.63 mg/g) in the yeast *Xanthophyllomyces dendrorhous* (*Phaffia rhodoxyma*) [22]. A similar result was reported for β-carotene content (0.35 to 0.66 mg/g) analysis in the red yeast *Rhodotorula glutinis* by FCM [39]. Freitas et al. also suggested that the auto-fluorescence measured in FL3 (>670 nm, excited with 488 nm light) was best correlated with the content of total carotenoids in the red yeast *Rhodosporidium toruloides* [40]. These foregoing results are contradictory to the existing knowledge that chlorophylls, particularly chlorophyll *a*, not carotenoids, emit auto-fluorescence at the maximum wavelength of nearly 680 nm when excited with 488 nm light [41]. We assumed that this might by caused by the low content of carotenoids, and their fluorescence was seriously interfered with other pigments such as carotenoid precursors, proteins, nucleotides, porphyrins, flavins, etc. [21,42]. In our present work, the astaxanthin content in *C. zofingiensis* cells reached 2.41 mg/g, which was significantly higher than that of previous studies. Our results indicated that the fluorescence in FL2 (580 nm) based on FCM was closely correlated with the content of cellular carotenoids, particularly astaxanthin, and FL3 (>675 nm) correlated directly with chlorophylls, which can be intuitively rationalized by the corresponding emissions of the respective pigments [35]. Kleinegris et al. reported that the 560 nm fluorescence of the green microalga *Dunaliella salina* was emitted by β-carotene (excitation at 488 nm). In *Symbiodinium*, the green fluorescence signal in FL1 (529 ± 28 nm band-pass filter) was generally used for β-carotene analysis [43]. These results demonstrated that the fluorescence intensities at the range of 530–580 nm had a close relationship with carotenoids, which was the foundation of the FCM method for astaxanthin analysis.

The Car/Chl ratio was previously demonstrated as the informative marker of cellular physiological stress in the microalga *H. pluvialis* under high light irradiation and *Parietochloris incisa* [44,45]. Moreover, the Car/Chl ratio correlated closely with the specific optical density in the blue-green region normalized to the red absorption maximum (OD_500_/OD_678_), which could be applied for the monitoring of carotenogenesis in *H. pluvialis* using optical sensors [12]. In this work, we explored the correlation of the Car/Chl ratio with the fluorescence intensities in FL1-FL3 channels using FCM. The Car/Chl ratio of mixotrophic *C. zofingiensis* increased substantially during culturing under stress conditions along with the accumulation of astaxanthin as well as the degradation of chlorophylls. Accordingly, the ratios of FL1/FL3 and FL2/FL3 increased along with the changes of pigments (Figure 7b), indicating a positive correlation between the Car/Chl ratio with the ratio of FL1/FL3 or FL2/FL3. Figure 9c,d shows that the Car/Chl ratio was linearly related with FL1/FL3 and FL2/FL3, though the coefficients of determination (*R*^2^) were 0.82. Therefore, the fluorescence intensities in FL1, FL2 and FL3 could also be applied to the assay of the Car/Chl ratio in *C. zofingiensis* cells.

To evaluate the determination of the astaxanthin content and Car/Chl ratio, we compared the values of the astaxanthin content and Car/Chl ratio measured by the FCM method with that measured by HPLC using correlation analysis according to the literature [46,47]. These correlation coefficients were 0.95 (*n* = 21, *p* < 0.01) and 0.91 (*n* = 21, *p* < 0.01), respectively, indicating that these different methods had a strong positive correlation with respect to measuring the astaxanthin content and Car/Chl ratio in *C. zofingiensis* cells. Furthermore, the FCM method can be further calibrated by HPLC through regression analysis to obtain more accurate values of the astaxanthin content and Car/Chl ratio. The regression curve for astaxanthin content determination was *Y* (astaxanthin content measured by FCM) = 0.8885 *X* (astaxanthin content measured by HPLC) + 0.1644 (*R*^2^ = 0.91). As for the Car/Chl ratio, the regression curve obtained by the similar procedures was *Y* (Car/Chl ratio measured by FCM) = 0.8278 *X* (Car/Chl ratio measured by HPLC) + 0.5383 (*R*^2^ = 0.83). However, our current work primarily aimed to examine the relationship between the fluorescence intensity and cellular astaxanthin content of algal cells and evaluate the reliability of the FCM method for predicting the astaxanthin content from the fluorescence intensity of algal cells. Thus, the FCM method without further calibration by HPLC was applied in the following method validation.

### 2.4. Linearity, Range, and Limits of Quantitation for the FCM Method

The FCM method was validated by the evaluation of its performance characteristics, such as linearity, precision, accuracy and recovery, and proved that it complied with established criteria for chemical analysis [48,49]. Through analyzing validation samples with different astaxanthin contents, we could demonstrate that the FCM method was capable of rapidly quantifying the astaxanthin content and Car/Chl ratio in living cells of mixotrophic *C. zofingiensis*. The best-fit linear regression of the astaxanthin content using HPLC versus MFI in FL2 using FCM was established through the coefficient of determination (*R*^2^ = 0.92) in this work. The lower limit of astaxanthin quantification (0.82 mg/g) corresponded to the minimum fluorescence intensity of 7988 by FCM (Figure 9b). The favorable range for accurate astaxanthin analysis based on FCM was between 0.82 and 2.41 mg/g dry cell weight (Figure 9b). Using HPLC analysis, the astaxanthin content in stress-induced *C. zofingiensis* was determined mainly in the range of 0.69–1.31 mg/g dry cell weight [10]. The approach we developed allows the direct monitoring of astaxanthin in *C. zofingiensis*. The method is thus suitable for the real-time monitoring of carotenogenesis in *C. zofingiensis* cells. In the above results (Figure 9c,d), the ratios of FL1/FL3 and FL2/FL3 were linearly correlated with the Car/Chl ratios, respectively, with the same coefficient of determination (*R*^2^ = 0.82). Considering the astaxanthin assay, the FL2/FL3 ratio was selected for the rapid estimation of the Car/Chl ratio in *C. zofingiensis* cells. The suitable range for the accurate determination of the Car/Chl ratio should be limited between 1.42 and 4.75 according to the regression.

### 2.5. Precision, Accuracy, and Recovery of FCM Method

The precision of the FCM method is documented in Table 1. The sample of *C. zofingiensis* cells with an astaxanthin content of 2.08 mg/g and Car/Chl ratio of 3.89 (determined by HPLC) was used for this evaluation. The mean astaxanthin content of 2.03 mg/g by FCM was very close to the true value based HPLC with a relative error (ER) of 1.93% and relative standard deviation (RSD) of 2.02% (*n* = 5), indicating the good precision of the FCM method. Similarly, the mean Car/Chl ratio of 4.32 based on FCM was also close to the value determined by HPLC with a relative error (ER) of 11.10% and relative standard deviation (RSD) of 1.10% (*n* = 5), indicating that this approach is feasible for rapidly estimating the Car/Chl ratio with a suitable precision.

In pharmaceutical analysis, recovery assays using spiked matrices and spiked samples are important tools for assessing the method accuracy [50], which is generally recognized as the difference between the measured mean values by the established method and the true values of validation samples [49]. In the current work, the accuracy and recovery of the FCM method were accordingly developed and modified according to the recovery analysis of spiked samples. In the following experiments, two sets of samples taken from the same stressed culture were measured by FCM followed by HPLC for accuracy analysis. Astaxanthin contents based on HPLC were 0.92 ± 0.12 and 1.65 ± 0.22 mg/g dry weight for these two samples, respectively. According to the results listed in Table 2, the astaxanthin content determined by FCM was basically consistent with that of HPLC with less than 12% of overall variation between the two methods. The Car/Chl ratio determined by FCM was equivalent to that determined by HPLC with ca. 3% overall variation. These results demonstrated that the FCM approach is feasible to determine the astaxanthin content and Car/Chl ratio in the suitable range and fulfills the requirements of the analytical methodology without sacrificing accuracy.

Additionally, the measured values of the samples subjected to FCM and HPLC were examined to assess the recovery analysis of the current method. As shown in Table 2, the average recovery of samples subjected to FCM for astaxanthin analysis was 102.89%, within the acceptable range of 80–120%. The relative standard deviation (RSD) of samples (*n* = 5) was as low as 2.04%, indicating the good repeatability in the recovery analysis of the FCM method. The average recovery of the Car/Chl ratio was relatively low owing to the weak correlation of FL2/FL3 vs. Car/Chl at a low carotenoid content. A reasonable explanation might be the variation of carotenoid composition and the steady aggregation of other carotenoids at different cultivation stages of mixotrophic *C. zofingiensis* [12]. Thus, we concluded that FCM, as a rapid and simple method, can be applied to the analysis of the astaxanthin content and Car/Chl ratio with acceptable precision and accuracy. It is worth noting that an obvious disadvantage of this method is that it requires instrumental determination under identical conditions and the pigment concentration in the linear range of the method. When cultivated in different systems and stress conditions, microalgae have a wide range of intercellular carotenoids and variable pigment composition that could be easily surveyed by the introduced FCM approach. Variations contributed to the non-linear relationship between the estimated pigments and the fluorescence intensities when using FCM. Furthermore, algal cells sampled from the cultures had to be analyzed by FCM directly and immediately under identical conditions to avoid environmental factors affecting the determination of the auto-fluorescence of algal cells. The system settings of FCM, for example, the event limit (total events collected), fluorescence detector voltages, and compensation controls, are also crucial for the determination of samples as well as the above-mentioned factors. These parameters of system settings must be identical in each assay of FCM to avoid technique errors. Taking together all the considerations, if the culture conditions of algal cells or the settings of FCM were changed, an FCM assay with a suitable regression equation should be re-established accordingly for the robustness of this approach in rapid estimation.

## 3. Materials and Methods

### 3.1. Microalgae and Culture Conditions

The green microalga *Chromochloris zofingiensis* (ATCC30412) was purchased from the American Type Culture Collection (ATCC, Rockville, MD, USA) and maintained at 4 °C on agar slants or plates of modified basal medium [51]. The cells were cultivated in the same liquid medium containing 10 g/L of glucose in shaking flasks at 26 °C, 150 r/m under continuous illumination with white fluorescence lights (ca. 10 μmol photon m^−2^ s^−1^). The culture reached the exponential phase after 96 h; then, we transferred 10 mL of the seed culture into 100 mL of modified basal medium containing 30 g/L of glucose and 0.6 g/L of NaNO_3_ in 250 mL Erlenmeyer flasks to generate a high C/N ratio of 200. The continuous light intensity was increased to 80 μmol photon m^−2^ s^−1^ for astaxanthin accumulation during the following 288 h. From 146 h of the stressed culture, the cells were sampled every 24 h and harvested by centrifugation at 3800 × *g* for 2 min. The biomass concentration was used to monitor the cell growth. The fluorescence intensities of living cells in FL1–FL3 were determined by FCM, while the pigment content in frozen dry biomass was analyzed by HPLC.

### 3.2. Chemicals and Reagents

HPLC-grade methanol and methyl *tert*-butyl ether (MTBE) were purchased from Thermo Fisher Scientific Inc. (Waltham, MA, USA). Astaxanthin, lutein, zeaxanthin, canthaxanthin, chlorophyll *a* and *b* as standards were purchased from Sigma Chemical Co. (St. Louis, MO, USA). Other chemicals and reagents of analytical grade were purchased from the local companies. Deionized water was purified by using a Milli-Q water system (Merck Millipore, Billerica, MA, USA) and filtered through a 0.22 μm membrane for HPLC analysis.

### 3.3. Emission Spectra of Fluorescence and Imaging by CLSM

The fluorescence emission spectra of *C. zofingiensis* cells were detected in the range from 500 nm to 800 nm using a fluorospectrophotometer (HITACHI F-7000, Hitachi, Tokyo, Japan) at an excitation wavelength of 488 nm.

A Leica TCS SP5X confocal laser scanning microscopy (CLSM, Leica Microsystems, Milton Keynes, UK) was used to capture the fluorescence images of mixotrophic cells of *C. zofingiensis*. Intracellular distribution of pigments in cells was viewed by scanning using a 488 nm argon laser, and light emission was recorded in the green (520–540 nm), yellow (570–590 nm), and red channels (670–730 nm) to monitor fluorescence signals of *C. zofingiensis* cells.

### 3.4. Flow Cytometry

Flow cytometry (model BD Accuri C6 Accuri Cytometers, Inc., Ann Arbor, MI, USA) was used for the cytometric analysis of *C. zofingiensis*. Two 50 mW air cooled lasers (exciting at 488 nm and 640 nm) and four interference filters (FL1 at 533 ± 15 nm, FL2 at 585 ± 20 nm, FL3 > 670 nm, and FL4 at 675 ± 25 nm) were used. The detailed procedure was modified according to a previous reference [22]. Briefly, the cells were centrifuged at 3800× *g* for 2 min and washed twice with potassium phosphate buffer (10 mM, pH 7.4). The cell resuspension was adjusted to 0.6–2 × 10^6^ cells/mL as the optimal cell density and then filtered through a membrane (400 mesh) before cytometric analysis.

In FCM analysis, according to the cell size of *C. zofingiensis*, the flow rate of injection was optimized to 35 μL/min, and 10,000 cells were count for analysis of each sample. The signals of forward scatter (FSC), side scatter (SSC), fluorescence intensities in four channels (FL1–FL4) were detected simultaneously. Data were further analyzed by Accuri^®^ CFlow software (Accuri Cytometers, Inc., Ann Arbor, MI, USA) and Flowjo software (FlowJo, LLC., Ashland, OR, USA) for statistical analysis. All samples were performed in triplicate.

### 3.5. HPLC Analysis

The lyophilized biomass was prepared using a freeze dryer (Modulyod-230, Thermo Scientific, Waltham, MA, USA). According to the literature [11] with a few minor modifications, the pigments were extracted by repeatedly adding methanol/dichloromethane (3:1, *v*/*v*) containing 0.1% (*w*/*v*) butylated hydroxytoluene (BHT) into the bead-beating tube until the biomass became colorless. The supernatant containing pigments was blow-dried by nitrogen gas and resolved in methanol/MTBE (1:1, *v*/*v*) containing 0.1% (*w*/*v*) BHT for HPLC analysis. The extraction process was performed under dim light, and the extract was kept at 4 °C.

A reversed-phase HPLC system (DIONEX P680, Thermo Scientific, Waltham, MA, USA) equipped with a Waters YMC™ Carotenoid C30 column (4.6 × 150 mm, 3 μm) and a photodiode array detector (PDA-100, Thermo Scientific, Waltham, MA, USA) was employed to separate and identify the pigments. The mobile phase consisted of methanol (A), MTBE (B), and deionized water (C) at a flow rate of 0.8 mL/min. A linear gradient program was employed as follows: 0–6 min, from 95% A/5% B to 80% A/20% B; 6–12 min, to 60% A/38% B/2% C; 12–28 min, to 50% A/48% B/2% C and isocratic for 5 min. A 20 μL sample was injected into the column, and the temperature was maintained at 30 °C. All peaks were scanned at 300–700 nm and identified by comparison with the retention times and the UV-vis absorption and spectral fine structures of the standards and information from the literature [11] followed by quantification based on the standard curves.

### 3.6. Correlation and Regression Analysis of the Astaxanthin Content *versus* Fluorescence Intensities

The correlation and regression analyses of astaxanthin content determined by HPLC and fluorescence intensities measured by FCM of algal cells were performed to explore their potential relationships. Initially, the values of fluorescence intensities (a.u.) in FL1 and FL2 and the astaxanthin content (μg/g, dry weight of algal cells) were logarithmically transformed to satisfy the homogeneity of variances assumption for the errors and to linearize the fit [21,52]. Then, Pearson’s correlation coefficients (*r*) between fluorescence intensities and astaxanthin contents were calculated by SPSS software (version 19.0, SPSS, Inc., Chicago, IL, USA) to measure the degree of association between these variables. If a strong correlation existed, the fluorescence intensities in FL1 and FL2 were plotted against the astaxanthin content of every sample, and regression analysis was performed to obtain the regression equations for predicting astaxanthin from fluorescence intensities based on FCM. With similar procedures, the correlations between Car/Chl ratios and FL1/FL3 or FL2/FL3 were assessed by Pearson's correlation coefficients (*r*), and the regression curves for predicting the Car/Chl ratios were constructed for the samples if strong correlations existed (i.e., Pearson’s correlation coefficients were higher than 0.8). The regression curves with the highest coefficients of determination (*R*^2^) were selected for predicting the astaxanthin content and Car/Chl ratio for the unknown samples through the fluorescence intensities measured by FCM under specific conditions. In order to avoid environmental preparation errors, algal cells sampled from the cultures should be analyzed by FCM instantly and directly. All system settings of FCM should be identical in each assay of each set of samples, or the values determined from the regression curve might be invalid. If the culture conditions of algal cells or the system settings of FCM are changed, the regression curve must be re-established accordingly to avoid technique errors in sample determination.

Moreover, the correlation between these two methods was determined based on the values of the astaxanthin content and Car/Chl ratio measured by FCM and HPLC according to the literature [46,47]. The correlation coefficients were calculated by SPSS 19.0 (SPSS, Inc., Chicago, IL, USA) and applied to estimate the correlation between these two methods. If a strong correlation was detected, regression analysis between the FCM and HPLC values was performed. This procedure served to explore the possibility that the FCM method can be further calibrated by HPLC to obtain more accurate values of the astaxanthin content and Car/Chl ratio.

### 3.7. Method Validation

The FCM method validation was performed from the analysis of validation samples to evaluate its performance characteristics and prove that it fulfilled the requirements of the established criteria for determination.

#### 3.7.1. Linear Range and Limits for FCM Method

The fluorescent intensities in FL1 and FL2 determined by FCM were plotted against the astaxanthin content to show the best-fit correlation, while the ratios of FL1/FL3 and FL2/FL3 were correlated with Car/Chl ratios in order to investigate their relationships. Based on these strong correlations, regression analysis was performed, and the regression curves with the highest coefficients of determination (*R*^2^) were selected for predicting the astaxanthin content and Car/Chl ratio of the unknown samples. The linear range and limits for the FCM method were obtained according to the regression curves.

#### 3.7.2. Precision

The living cells of *C. zofingiensis* in mixotrophic growth were directly analyzed in vivo immediately by FCM after sampling to avoid the potential degradation of astaxanthin. The precision of the FCM method was assessed by analysis of five technical replicates from the same culture and represented as the relative error (RE) and relative standard deviation (RSD).

#### 3.7.3. Accuracy and Recovery

Accuracy, generally recognized as the difference between the measured mean values by the established method and the true values of validation samples [49], can be estimated from recovery tests using samples spiked with a known concentration of the analyte [50]. The accuracy assessment of the FCM method in our study was accordingly developed and modified to this approach. In brief, we conducted a similar study assessing the accuracy by the use of spiked samples. Two sets of cells taken from the same stressed culture with either high or low astaxanthin content were detected by FCM and HPLC methods to ensure the accuracy, which was represented as the percentage of the mean or true value variation. The recovery was carried out from the two sets of cells similar to spiked samples. The acceptable recovery value should be between 80% and 120%.

## 4. Conclusions

The fluorescence signals and pigment contents in mixotrophic *C. zofingiensis* cells during cultivation were analyzed by FCM and HPLC. A positive linear regression between the auto-fluorescence of cells based on FCM and the accurate content of astaxanthin or the Car/Chl ratio based on HPLC was established. The method introduced is suitable as a screening procedure complementary to HPLC. The FCM approach based on the auto-fluorescence has the advantages of simplicity and speed by comparison with the traditional HPLC assay. This FCM method can thus complement the traditional assay of pigments and provides a rapid and efficient approach for on-site monitoring of stress-induced carotenogenesis during the cultivation of *C. zofingiensis*. The FCM method developed in the current work is reliable and affordable for the rapid estimation of carotenoids, particularly astaxanthin, in *C. zofingiensis* cells with an acceptable precision. It is comparable and competitive to established rapid methods for carotenoid analysis. Our study demonstrates that flow cytometry with suitable algorithms, after calibration by HPLC data, is a powerful tool for studies of carotenogenesis and for physiological characterization of microalgae. The method is especially recommended in processes that require high-throughput methods for the rapid analysis and real-time monitoring of cellular pigments in *C. zofingiensis* under stress conditions.

## Figures and Tables

**Table 1:** The precision of the FCM method for the rapid quantification of astaxanthin and the Car/Chl ratio.

No.	MFI in FL2 (a.u.)	MFI in FL3 (a.u.)	Asta. Content by FCM (mg/g)	Car/Chl Ratio by FCM	Astaxanthin			Car/Chl Ratio		
					Mean Value (mg/g)	Relative Error (RE, %)	Relative Standard Deviation (RSD, %)	Mean Value	Relative Error (RE, %)	Relative Standard Deviation (RSD, %)
1	14,230	468,000	1.99	4.37	2.03 ± 0.05	1.93	2.20	4.32 ± 0.04	11.05	1.10
2	14,350	468,430	2.01	4.36						
3	14,747	489,000	2.10	4.30						
4	14,630	484,860	2.07	4.30						
5	14,405	480,100	2.02	4.28						

No. Five independent samples were taken from two independent culturing flasks. MFI: mean fluorescence intensity; FL2: 585 nm ± 20 nm; FL3: >670 nm; a.u.: arbitrary unit; Asta: astaxanthin; Car/Chl ratio: the carotenoid-to-chlorophyll ratio; a sample of *C. zofingiensis* cells with astaxanthin content of 2.08 mg/g and Car/Chl of 3.89 (determined by HPLC) was used for the evaluation.

**Table 2:** The accuracy and recovery of the FCM method for the rapid quantification of astaxanthin and the Car/Chl ratio.

No.		1	2	3	4	5	Mean Value
**Sample 1**	MFI in FL2 (a.u.)	7914	8056	8301	8233	8117	8124 ± 151
	MFI in FL3 (a.u.)	634,600	651,000	726,080	718,860	704,560	687,000 ± 41,500
	FL2/FL3	0.0125	0.0124	0.0114	0.0115	0.0115	0.0119 ± 0.0005
**Measured value by FCM**	Asta. (mg/g)	0.81	0.83	0.87	0.86	0.84	0.84 ± 0.02
	Car/Chl	1.83	1.82	1.69	1.69	1.70	1.75 ± 0.07
**Accuracy analysis (%)**	Asta.	87.75	90.17	94.41	93.23	91.22	91.36 ± 2.61
	Car/Chl	79.15	78.57	72.92	73.03	73.44	75.42 ± 3.15
**Sample 2**	MFI in FL2 (a.u.)	12,177	12,281	12,545	12,408	12,186	12,319 ± 156
	MFI in FL3 (a.u.)	558,740	565,610	598,240	591,460	580,000	578,800 ± 16,700
	FL2/FL3	0.0218	0.0217	0.0209	0.0209	0.0210	0.0213 ± 0.0005
**Measured value by FCM**	Asta.(mg/g)	1.56	1.58	1.64	1.61	1.57	1.59 ± 0.03
	Car/Chl	3.13	3.12	3.02	3.02	3.02	3.06 ± 0.06
**Accuracy analysis (%)**	Asta.	94.75	95.99	99.17	97.52	94.86	96.46 ± 1.88
	Car/Chl	103.65	103.28	99.86	99.90	100.00	101.34 ± .95
**Recovery (%)**	Asta.	103.58	103.33	105.16	102.93	99.45	
	Car/Chl	78.60	78.73	80.41	80.31	80.02	
**Average recovery (%)**	Asta.			102.89 ± 2.10			
	Car/Chl			79.61 ± 0.88			
**Relative standard deviation of recovery (RSD, %)**	Asta.			2.04			
	Car/Chl			1.10			

MFI: mean fluorescence intensity; FL2: 585 nm ± 20 nm; FL3: >670 nm; a.u.: arbitrary unit; Asta.: astaxanthin; Car/Chl ratio: the carotenoid-to-chlorophyll ratio; Sample 1: *C. zofingiensis* cells with astaxanthin content of 0.92 mg/g and Car/Chl of 2.32 (determined by HPLC); Sample 2: *C. zofingiensis* cells with astaxanthin content of 1.65 mg/g and Car/Chl of 3.02 (determined by HPLC).

## References

[1] Ambati R.R., Phang S.M., Ravi S., Aswathanarayana R.G. (2014). Astaxanthin: Sources, extraction, stability, biological activities and its commercial applications-a review. Mar. Drugs.

[2] Fassett R.G., Coombes J.S. (2011). Astaxanthin: A Potential Therapeutic Agent in Cardiovascular Disease. Mar. Drugs.

[3] Wu H., Niu H., Shao A., Wu C., Dixon B.J., Zhang J., Yang S., Wang Y. (2015). Astaxanthin as a potential neuroprotective agent for neurological diseases. Mar. Drugs.

[4] Higuera-Ciapara I., Felix-Valenzuela L., Goycoolea F.M. (2006). Astaxanthin: A review of its chemistry and applications. Crit. Rev. Food Sci. Nutr..

[5] Schmidt I., Schewe H., Gassel S., Jin C., Buckingham J., Huembelin M., Sandmann G., Schrader J. (2011). Biotechnological production of astaxanthin with *Phaffia rhodozyma*/*Xanthophyllomyces* dendrorhous. Appl. Microbiol. Biotechnol..

[6] Huangfu J., Liu J., Sun Z., Wang M., Jiang Y., Chen Z.-Y., Chen F. (2013). Antiaging effects of astaxanthin-rich alga *Haematococcus pluvialis* on fruit flies under oxidative stress. J. Agric. Food Chem..

[7] Guerin M., Huntley M.E., Olaizola M. (2003). Haematococcus astaxanthin: Applications for human health and nutrition. Trends Biotechnol..

[8] Champenois J., Marfaing H., Pierre R. (2015). Review of the taxonomic revision of *Chlorella* and consequences for its food uses in Europe. J. Appl. Phycol..

[9] Solovchenko A.E. (2015). Recent breakthroughs in the biology of astaxanthin accumulation by microalgal cell. Photosynthesis Res..

[10] Liu J., Sun Z., Gerken H., Liu Z., Jiang Y., Chen F. (2014). *Chlorella zofingiensis* as an Alternative Microalgal Producer of Astaxanthin: Biology and Industrial Potential. Mar. Drugs.

[11] Mulders K.J.M., Weesepoel Y., Bodenes P., Lamers P.P., Vincken J.-P., Martens D.E., Gruppen H., Wijffels R.H. (2015). Nitrogen-depleted *Chlorella zofingiensis* produces astaxanthin, ketolutein and their fatty acid esters: A carotenoid metabolism study. J. Appl. Phycol..

[12] Solovchenko A., Aflalo C., Lukyanov A., Boussiba S. (2013). Nondestructive monitoring of carotenogenesis in Haematococcus pluvialis via whole-cell optical density spectra. Appl. Microbiol. Biotechnol..

[13] Kim D.-Y., Vijayan D., Praveenkumar R., Han J.-I., Lee K., Park J.-Y., Chang W.-S., Lee J.-S., Oh Y.-K. (2016). Cell-wall disruption and lipid/astaxanthin extraction from microalgae: *Chlorella* and *Haematococcus*. Bioresour. Technol..

[14] Liu J., Sun Z., Gerken H., Huang J., Jiang Y., Chen F. (2014). Genetic engineering of the green alga *Chlorella zofingiensis*: A modified norflurazon-resistant phytoene desaturase gene as a dominant selectable marker. Appl. Microbiol. Biotechnol..

[15] Amorim-Carrilho K.T., Cepeda A., Fente C., Regal P. (2014). Review of methods for analysis of carotenoids. Trac-Trends Anal. Chem..

[16] Rivera S.M., Canela-Garayoa R. (2012). Analytical tools for the analysis of carotenoids in diverse materials. J. Chromatogr..

[17] Nicolaie B.M., Beullens K., Bobelyn E., Peirs A., Saeys W., Theron K.I., Lammertyn J. (2007). Nondestructive measurement of fruit and vegetable quality by means of NIR spectroscopy: A review. Postharvest Biol. Technol..

[18] De Oliveira V.E., Neves Miranda M.A.C., Silva Soares M.C., Edwards H.G.M., Cappa de Oliveira L.F. (2015). Study of carotenoids in cyanobacteria by Raman spectroscopy. Spectrochim. Acta Part A-Mol. Biomol. Spectrosc..

[19] Rungpichayapichet P., Mahayothee B., Khuwijitjaru P., Nagle M., Mueller J. (2015). Non-destructive determination of beta-carotene content in mango by near-infrared spectroscopy compared with colorimetric measurements. J. Food Compost. Anal..

[20] Hyka P., Lickova S., Pribyl P., Melzoch K., Kovar K. (2013). Flow cytometry for the development of biotechnological processes with microalgae. Biotechnol. Adv..

[21] An G.H., Suh O.S., Kwon H.C., Kim K., Johnson E.A. (2000). Quantification of carotenoids in cells of *Phaffia rhodozyma* by autofluorescence. Biotechnol. Lett..

[22] Ukibe K., Katsuragi T., Tani Y., Takagi H. (2008). Efficient screening for astaxanthin-overproducing mutants of the yeast *Xanthophyllomyces dendrorhous* by flow cytometry. FEMS Microbiol. Lett..

[23] An G.H., Bielich J., Auerbach R., Johnson E.A. (1991). Isolation and characterization of carotenoid hyperproducing mutants of yeast by flow cytometry and cell sorting. Biotechnology.

[24] Cordero B.F., Couso I., Leon R., Rodriguez H., Angeles Vargas M. (2012). Isolation and Characterization of a Lycopene epsilon-Cyclase Gene of Chlorella (Chromochloris) zofingiensis. Regulation of the Carotenogenic Pathway by Nitrogen and Light. Mar. Drugs.

[25] Demmig-Adams B., Gilmore A.M., Adams W.W. (1996). Carotenoids 3: In vivo function of carotenoids in higher plants. FASEB J..

[26] Gabriela Lagorio M., Cordon G.B., Iriel A. (2015). Reviewing the relevance of fluorescence in biological systems. Photochem. Photobiol. Sci..

[27] Kleinegris D.M.M., van Es M.A., Janssen M., Brandenburg W.A., Wijffels R.H. (2010). Carotenoid fluorescence in *Dunaliella salina*. J. Appl. Phycol..

[28] Ermakov I.V., Sharifzadeh M., Ermakova M., Gellermann W. (2005). Resonance Raman detection of carotenoid antioxidants in living human tissue. J. Biomed. Opt..

[29] Rioboo C., Gonzalez-Barreiro O., Abalde J., Cid A. (2011). Flow cytometric analysis of the encystment process induced by paraquat exposure in *Haematococcus pluvialis* (Chlorophyceae). Eur. J. Phycol..

[30] Wayama M., Ota S., Matsuura H., Nango N., Hirata A., Kawano S. (2013). Three-dimensional ultrastructural study of oil and astaxanthin accumulation during encystment in the green alga Haematococcus pluvialis. PLoS ONE.

[31] Schoefs B. (2002). Chlorophyll and carotenoid analysis in food products. Properties of the pigments and methods of analysis. Trends Food Sci. Technol..

[32] Englert G., Britton G., Liaaenjensen S., Pfander H. (1995). UV/Visible spectroscopy. Carotenoids; Volume 1B: Spectroscopy.

[33] Porcar-Castell A., Berry J.A. (2014). Linking chlorophyll a fluorescence to photosynthesis for remote sensing applications: Mechanisms and challenges. JEXB.

[34] Narayan A., Misra M., Singh R. (2012). Chlorophyll Fluorescence in Plant Biology. Biophysics.

[35] Hu Z., Li Y., Sommerfeld M., Chen F., Hu Q. (2008). Enhanced protection against oxidative stress in an astaxanthin-overproduction *Haematococcus mutant* (Chlorophyceae). Eur. J. Phycol..

[36] Davis R.W., Carvalho B.J., Jones H.D.T., Singh S. (2015). The role of photo-osmotic adaptation in semi-continuous culture and lipid particle release from Dunaliella viridis. J. Appl. Phycol..

[37] Eullaffroy P., Vernet G. (2003). The F684/F735 chlorophyll fluorescence ratio: A potential tool for rapid detection and determination of herbicide phytotoxicity in algae. Water Res..

[38] Chen T., Liu J., Guo B., Ma X., Sun P., Liu B., Chen F. (2015). Light attenuates lipid accumulation while enhancing cell proliferation and starch synthesis in the glucose-fed oleaginous microalga *Chlorella Zofingiensis*. Sci. Rep..

[39] Cutzu R., Clemente A., Reis A., Nobre B., Mannazzu I., Roseiro J., da Silva T.L. (2013). Assessment of beta-carotene content, cell physiology and morphology of the yellow yeast *Rhodotorula glutinis* mutant 400A15 using flow cytometry. J. Ind. Microbiol. Biotechnol..

[40] Freitas C., Nobre B., Gouveia L., Roseiro J., Reis A., da Silva T.L. (2014). New at-line flow cytometric protocols for determining carotenoid content and cell viability during Rhodosporidium toruloides NCYC 921 batch growth. Process Biochem..

[41] Kula M., Rys M., Mozdzen K., Skoczowski A. (2014). Metabolic activity, the chemical composition of biomass and photosynthetic activity of Chlorella vulgaris under different light spectra in photobioreactors. Eng. Life Sci..

[42] Mueller S., Galliardt H., Schneider J., Barisas B.G., Seidel T. (2013). Quantification of Forster resonance energy transfer by monitoring sensitized emission in living plant cells. Front. Plant Sci..

[43] Lee C.S., Yeo Y.S.W., Sin T.M. (2012). Bleaching response of Symbiodinium (zooxanthellae): Determination by flow cytometry. Cytom. Part A.

[44] Solovchenko A.E., Chivkunova O.B., Maslova I.P. (2011). Pigment composition, optical properties, and resistance to photodamage of the microalga Haematococcus pluvialis cultivated under high light. Russ. J. Plant Physiol..

[45] Solovchenko A.E., Khozin-Goldberg I., Cohen Z., Merzlyak M.N. (2009). Carotenoid-to-chlorophyll ratio as a proxy for assay of total fatty acids and arachidonic acid content in the green microalga Parietochloris incisa. J. Appl. Phycol..

[46] Lee J., Rennaker C., Wrolstad R.E. (2008). Correlation of two anthocyanin quantification methods: HPLC and spectrophotometric methods. Food Chem..

[47] Lao F., Giusti M.M. (2016). Quantification of Purple Corn (*Zea mays* L.) Anthocyanins Using Spectrophotometric and HPLC Approaches: Method Comparison and Correlation. Food Anal. Methods.

[48] Taverniers I., De Loose M., Van Bockstaele E. (2004). Trends in quality in the analytical laboratory. II. Analytical method validation and quality assurance. Trac-Trends Anal. Chem..

[49] Peters F.T., Drummer O.H., Musshoff F. (2007). Validation of new methods. Forensic Sci. Int..

[50] Gonzalez A.G., Herrador M.A., Asuero A.G. (1999). Intra-laboratory testing of method accuracy from recovery assays. Talanta.

[51] Chen J., Liu X., Wei D., Chen G. (2015). High yields of fatty acid and neutral lipid production from cassava bagasse hydrolysate (CBH) by heterotrophic Chlorella protothecoides. Bioresour. Technol..

[52] Asuero A.G., Bueno J.M. (2011). Fitting Straight Lines with Replicated Observations by Linear Regression. IV. Transforming Data. Crit. Rev. Anal. Chem..

